# Need for a standardized protocol for stress echocardiography in provoking subaortic and valvular gradient in various cardiac conditions

**DOI:** 10.1186/1476-7120-12-26

**Published:** 2014-07-14

**Authors:** Pawel Petkow Dimitrow, Carlos Cotrim, Tsung O Cheng

**Affiliations:** 1 2nd Department of Cardiology CMUJ, 31-501 Cracow, Poland; 2 Circulation Department, Hospital da Cruz Vermelha Portuguesa, Lisboa, Portugal; 3 Department of Medicine, The George Washington University Medical Center, 2150 Pennsylvania Avenue, NW, Washington, DC 20037, USA

**Keywords:** Exercise, Echocardiography, Stress test, Upright, Hypertrophic cardiomyopathy, valve stenosis

## Abstract

(Semi) supine exercise testing has an established role in the evaluation of patients with valvular heart disease and can help clinical decision making. Stress echocardiography has the advantages of its wide availability, low cost, and versatility for the assessment of disease severity. However, exercise-induced changes in valve hemodynamics, left ventricular outflow obstruction and pulmonary artery pressure depended on load variation. Changing position from supine to upright rapidly decreases load conditions for the ventricles. Therefore several cardiac centers have proposed exercise stress echocardiography in the upright position with gradient monitoring sometimes also in post-exercise recovery. Doppler measurement of subaortic gradient has been a very helpful and informative examination in several heart diseases (especially in hypertrophic cardiomyopathy, valve heart diseases, prosthesis dysfunction).

## Introduction

Stress echocardiography has been introduced many years ago as a valuable method in the detection of myocardial ischemia in patients with known or suspected coronary artery disease by assessing wall motion abnormalities [1]. At least in some centers two-dimensional stress echocardiography is performed in the standing position throughout the exercise stress test with image acquisition at peak exercise [2]. Standing position, both at rest and during exercise, is a normal and fundamental activity of daily life, but may precipitate an unexpected fall in cardiac patients predisposed to syncope, especially in patients with unsuspected aortic or sub-aortic obstruction. Stress echocardiography is not only useful for diagnosis of coronary artery disease but also for Doppler measurement of sub-aortic valvular pressure gradient.

The evaluation of sub-aortic obstruction only at rest might underestimate the full impact of the lesion and its clinical effects. In a pioneering study published in 1966, Mason, Braunwald and Ross [3] reported that cardiac symptoms in these patients were noted most commonly when they were in the erect position, and these symptoms also tended to occur during or immediately after exertion.

### Exercise test protocol

The exercise protocol is a complex issue. Currently there are 3 protocols used for stress echocardiography in provoking or exacerbating left ventricular outflow tract gradient (LVOTG) in patients with hypertrophic cardiomyopathy (HCM):

1. Fully-physiological: upright position during both exercise and recovery with continuous echocardiographic monitoring of LVOTG (recommended in our opinion)

2. Non-physiological: supine position at both stages with echocardiographic monitoring

3. Semi-physiological: treadmill exercise followed by echocardiographic recording at recovery in a supine position.

Supine exercise is technically less demanding but also less physiological than upright exercise. It should be underscored that orthostatic exercise reflects physical exercise during everyday activity and reduces the preload more than supine exercise.

Recently, Lafitte et al. [4] clearly documented that the 2^nd^ approach could not be considered to be a pure evaluation of exercise dynamics and also postulated that 3^rd^ option is not adequate because dramatic pre-load variations were observed a few seconds after the end of exercise. They [4] further stressed that, when the subject was upright at the termination of exercise, there was a large decrease in venous blood return to the heart, yielding a decreased left ventricular volume, a decreased wall stress, a continued sympathetic drive, and a hyperkinetic state like that observed during dobutamine-induced stress.

In the two most recent publications, Wittlieb-Weber et al. [5,6] stated that, since upright positioning was more physiologic, it seems logical that this would be the standard approach for LVOTG assessment. According to these authors, since increasingly more studies had been, and would be, published in evaluating the LVOTG by exercise stress echocardiography, standardization of this measurement and specific guidelines on stress echocardiography for this indication, which thus far had been lacking, should be stipulated.

### Methodology

**Scheme 1:**
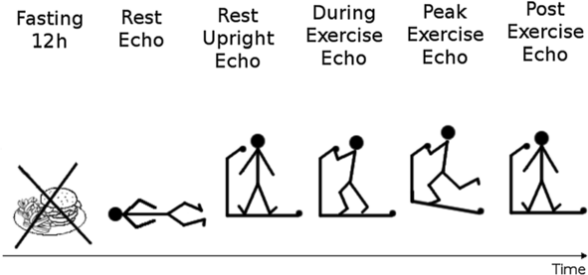
Graphic illustration of exercise protocol.

**Scheme 2:**
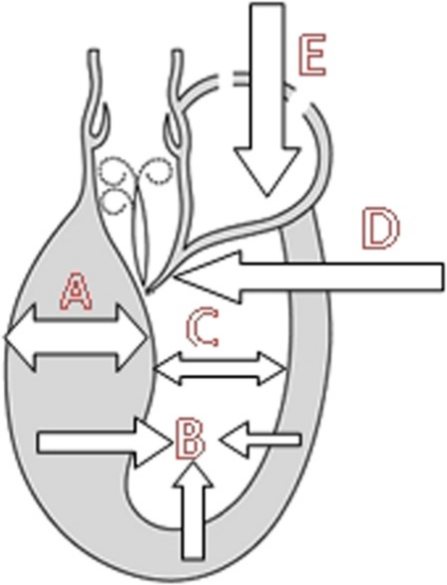
**Mechanisms predisposing to LVOTG induction.** A-Left ventricular hypertrophy – particularly basal septal segment (HCM, hypertension, storage disease). B-LV hypercontractivility (moderate tachycardia). C-Small size LV cavity (HCM, children, women, dehydratation). D-Prolonged/Thickened mitral leaflet(s). E-Reduced LV preload (dehydratation, diuretics, vasodilators, hemodialysis, fever, septic shock).

The specific preparation for exercise test for echocardiographic monitoring has been described in detail by Cotrim et al. [2]. Also, the echocardiographic technology in upright position has been precisely demonstrated in this publications. At the present moment, we would like to propose a standardized stress echocardiographic protocol for LVOTG provocation in HCM in accordance with several previous studies exploring not only HCM but also other cardiac conditions (Table 1, Scheme 1). Mechanisms predisposing to LVOT induction are summarized in Scheme 2. The Doppler-echocardiographic approach is from the apical view.

**Table 1:** Proposal of Echo-Doppler exercise echocardiography

**Protocol**	**Comments, references**
1. Period of 12-h fasting before exercise	[7]
2. Physiological exercise test for LVOTG provocation	
A/ Pre-exercise stage – echocardiography first in supine position and then in upright position	In some patients rapid emergence or increase in LVOTG has been reported after mere standing prior to orthostatic stress testing; under such circumstances, exercise provocation is not only unnecessary but even contraindicated, because of the risk of syncope.
B/ Position during exercise – upright	
C/ Exercise gradient monitoring – continuous	
D/ Moment of gradient measurement by Doppler echocardiography – peak exercise	
E/ Mode of exercise – treadmill	bicycle can be confidently used to acquire peak images, but peak O_2_ consumption is lower than that achieved by treadmill, which might lead to lower sensitivity. In addition, in contrast to treadmill, leg discomfort or lack of leg strength is a common reason for terminating prematurely the bicycling test.
F/ Termination of test – symptom-limited;	alternatively, pre-specified heart rate/workload (or combination).
G/ Post-exercise recovery – continuous monitoring of LVOTG in upright position	
Full protocol in treadmill 2A-G [8-13]	

### Investigation centers/cardiac diseases

We have performed systematic search across the publication database PubMed using combination of keywords: “echocardiography”, “exercise”, gradient”. We came across (as of May 12–15, 2014) 468 adequate publications concerning body position during exercise and moments of Doppler measurements (minimal including criteria for analysis presented in Table 2 must fulfill at a minimum items B and D proposed in Table 1).

**Table 2:** Doppler echo exercise upright treadmill/bicycle test – examples of some centers and examined diseases/conditions

**Country, town of center, type of exercise machine**	**Measured gradient**	**Clinical impact of exercise echo in UPRIGHT position**
1. Portugal, Almada , treadmill		Stratification risk of sudden death in HCM because LVOTG is risk factor
- Hypertrophic cardiomyopathy [8,14],	LVOT	Assessment of pharmacological and nonpharmacological methods reducing LVOTG
-Symptomatic athletes (Intra-ventricular obstruction induced by exercise in athletes with “positive screening” in medical evaluation for sports practice, [9]	LVOT	Verification of exercise-induced symptoms in relation to LVOTG
- Cardiac syndrome X [10]	LVOT	Monitoring treatment with beta blockers
- Pulmonary artery hypertension [11]	Transtricuspid	Verification of exercise-induced symptoms in relation to LVOTG
- Mitral stenosis [12]	Tranmitral	Monitoring the use of beta blockers
-Aortic stenosis [13]	Transaortic	Verification of exercise-increase of pulmonary hypertension (assessment of dyspnea)
		Verification of exercise-induced symptoms in relation to trasmitral gradient
		Verification of exercise-induced symptoms in relation to transaortic gradient
2. Poland, Cracow, treadmill		
- Hypertrophic cardiomyopathy [15,16]	LVOT	HCM see above
3. USA, Philadelphia, treadmill		
- “healthy“ youth for cardiac evaluation due palpitations, syndrome WPW, short of breath, chest pain [5]	LVOT	Hemodynamic verification of symptoms pathophysiology
4. Sweden, Vasteras, bicycle		
- Healthy athletes males [17]	Transaortic	Definition of normal value
5. USA, Spriengfield/ England, Liverpool, bicycle		
- Healthy adolescent boys and girls [18]	Transaortic	Definition of normal value
6. England, London, bicycle		
- Hypertrophic cardiomyopathy [19]	LVOT	HCM see above
- Anderson-Fabry disease [20]	LVOT	Hemodynamic assessment the effect of myocardial hypertrophy induced by storage disease
7. Spain, A Coruna, treadmill		
- Hypertrophic cardiomyopathy [21]	LVOT	HCM see above
8. Spain, Malaga, treadmill		
- Effort angina [22]	LVOT	Verification of exercise-induced symptoms
		Monitoring the effect of beta blockers
9. Altavilla Vicentina, Italy, treadmill (comparison with semi physiological test protocol)	LVOT	HCM see above
- Hypertrophic cardiomyopathy [23]		
10. Germany, Munster, bicycle	LVOT	HCM see above
- Hypertrophic cardiomyopathy [24]		
11. Korea, Seoul, treadmill	Transaortic	Verification of exercise-induced symptoms in relation to transaortic gradient
-Aortic stenosis [25]		
12. USA, Cleveland , treadmill	LVOT	HCM see above
-Hypertrophic cardiomyopathy [26]		
13. Canada, Quebec, bicycle	Transprothesis- Aortic	Assessment of prosthesis function and ‘patients-prosthesis mismatch’ phenomenon
- Patients with a bioprosthesis in the aortic valve position [27] , bicycle		
- Comparison of stentless versus stented bioprostheses in aortic valvular position [28], bicycle		

In transaortic valve level, normal values for healthy subjects during upright bicycle exercise in 24 adult healthy male endurance athletes from rest to peak exercise (at a heart rate of 160 bpm) are as follows:, the maximum aortic flow velocity almost doubled (1,14 vs 2,20 m/s) and the maximum transmitral flow velocity more than doubled (0,62 vs 1,43 m/s) [17]. The transaortic velocities with the similar increases were achieved in untrained adolescent boys (1,36 vs 2,08 m/s) and girls (1,08 vs 1,96 m/s) [18]. Maximal upright exercise transaortic gradient should be peaked below 20 mmHg in bicycle exercise.

**Figure 1:**
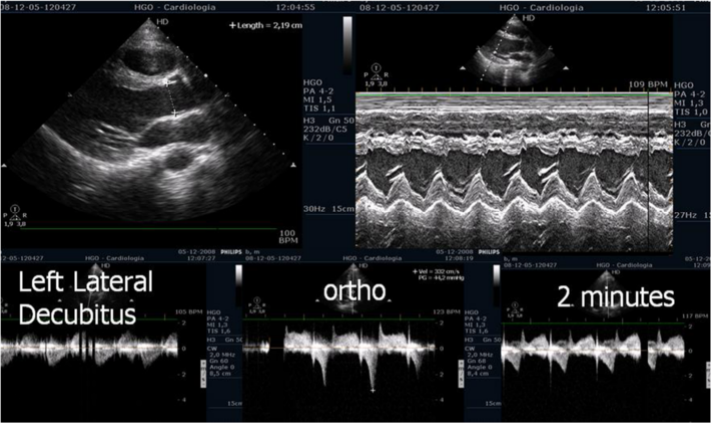
Echocardiogram before exercise with symptomatic athlete in left lateral decubitus position and in orthostatic position before and at beginning of exercise.

**Figure 2:**
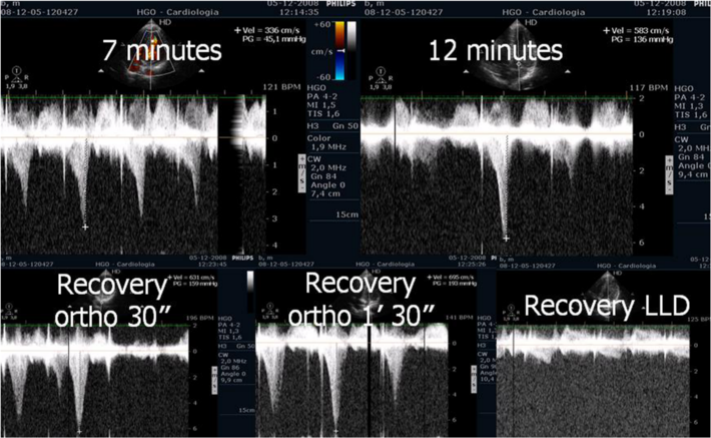
Intraventricular gradient in the various phases of exercise in same symptomatic athlete.

**Figure 3:**
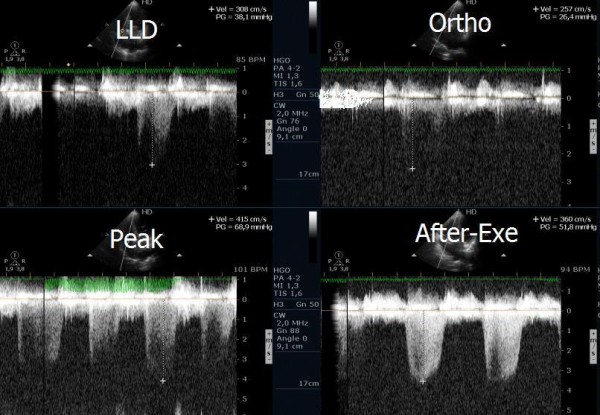
Right ventrícle /Right atrium gradient at different stages of the study in a patient with mitral stenosis.

**Figure 4:**
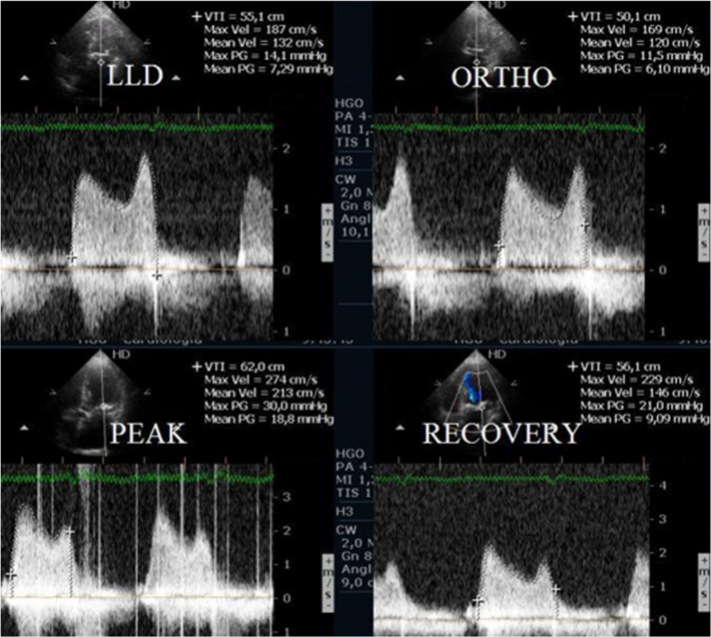
Left atrium/ left ventricle mean gradient, evaluated with CW Doppler, at different stages of the study in one patient with mitral stenosis.

**Figure 5:**
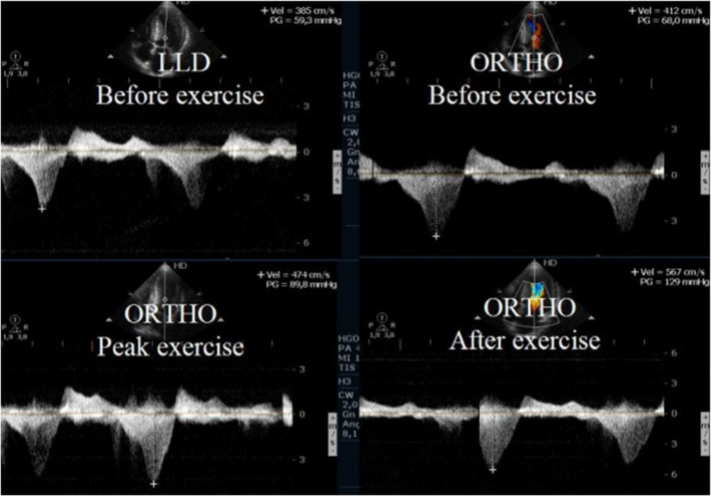
Intra-ventricular gradient present in all phases of the study in a HCM patient with increase also after exercise in orthostatic position.

We would like to emphasize that (sub)valvular gradient measurement during and after exercise may be diagnostically useful also in other diseases/conditions than HCM. Therefore, a standardized exercise protocol is of paramount importance for universal application in the practice of cardiology (Table 2), (Figures 1, 2, 3, 4 and 5), (Additional file 1, Additional file 2, and Additional file 3). In Table 2, we have included studies with at least Items B and D from Table 1. A significant number of studies on upright bicycle exercise was used; thus our analysis is heterogeneous. Items 1 and 2 in Table 2 described our own experiences.

### Limitation of method and learning curves

Elderly patients may not exercise well in supine positions [29]. On the other hand, treadmill exercise may predispose to syncope [30]; however, particularly in patients with a history of syncope, we should assess hemodynamic changes during treadmill exercise as potential risk factors. An additional advantage of treadmill exercise is the fact that patients usually can achieve higher workload.

The main limitation of exercise stress echocardiography is the presence of a poor acoustic window in some patients [31,32]. Furthermore, only apical view is imaginable. However, in Doppler-gradient examination feasibility is high and success rate may be achieved in more than 90% of the cases. The subvalvular LVOT gradient is easier to measure than the transvalvular gradient in aortic valvular stenosis [33]. In some clinical situation (hemodialysis room) only passive (non-exercise) orthostatic test may be applicable [34].

Imaging acquisition during exercise echocardiography is more difficult than during pharmacological stress, due to the greater increase in both heart and respiratory rates with exercise. Pharmacological stress echocardiography requires less skills than exercise stress echocardiography. On the other hand, most physicians and fellows in training acquire the necessary expertise to perform peak exercise studies on a treadmill with confidence after 100 cases.

### Advantages of method

The low cost, safety [35], diagnostic accuracy, possibility of evaluation of functional capacity and lack of radiation exposure should make exercise stress echocardiography an attractive procedure for patients with hypertrophic subvalvular or valvular aortic stenosis. The possibility of evaluation of Doppler data during and after exercise in orthostatic position in patients with LVOT obstruction provides very practical and useful information. Despite its limitations we believe that this test when standardized is suitable for use in every institution around the world.

## Conclusions

Doppler gradient measurement during, and symptomatic responses to, exercise provides the clinicians with important diagnostic and prognostic information that can contribute to subsequent clinical decisions and management [30,36].

Standing should be recommended as a physiologic provocative maneuver. In some patients, standing may guide therapy; in others, the exercise gradient measured in standing position provides a correct appreciation of the range of physiologically experienced gradients during normal daily upright activity.

Doppler echocardiography during and after upright exercise increases both the quality and quantity of information obtained in not only HCM but also many other clinical conditions. The preference of upright position is confirmed by positive experiences in many echocardiographic laboratories around the world.

## Abbreviations

LVOTG: Left ventricular outflow tract gradient; HCM: Hypertrophic cardiomyopathy.

## Competing interests

The authors declare that they have no competing interests.

## Authors’ contributions

All Authors have been involved in drafting the manuscript or revising it critically for important intellectual content and have given final approval of the version to be published.

## Supplementary Material

Additional file 1 Apical four-chamber view obtained in apical window before exercise in one patient with “non obstructive” HCM containing two dimensional data (with SAM). Click here for file

Additional file 2 Apical four-chamber view obtained in apical window at peak exercise in the same patient with SAM causing severe obstruction. Click here for file

Additional file 3 Apical four-chamber view obtained in apical window after exercise in the same patient with SAM causing severe obstruction. Click here for file
